# A Comprehensive View on the Protein Functions of Porcine Epidemic Diarrhea Virus

**DOI:** 10.3390/genes15020165

**Published:** 2024-01-26

**Authors:** Xin Li, Yiwan Wu, Zhibin Yan, Gen Li, Jun Luo, Shile Huang, Xiaofeng Guo

**Affiliations:** 1 College of Veterinary Medicine, South China Agricultural University, Guangzhou 510642, China; xinlilee@foxmail.com (X.L.); yiwan_wu515@163.com (Y.W.); 18826271402@163.com (Z.Y.); ligen223@126.com (G.L.); junluo@scau.edu.cn (J.L.); 2 Zhaoqing Branch Center of Guangdong Laboratory for Lingnan Modern Agricultural Science and Technology, Zhaoqing 526238, China; 3 Department of Biochemistry and Molecular Biology, Louisiana State University Health Sciences Center, 1501 Kings Highway, Shreveport, LA 71130-3932, USA; 4 Department of Hematology and Oncology, Louisiana State University Health Sciences Center, 1501 Kings Highway, Shreveport, LA 71130-3932, USA; 5 Feist-Weiller Cancer Center, Louisiana State University Health Sciences Center, 1501 Kings Highway, Shreveport, LA 71130-3932, USA

**Keywords:** porcine epidemic diarrhea virus (PEDV), protein function, viral proteins, innate immunity

## Abstract

Porcine epidemic diarrhea (PED) virus (PEDV) is one of the main pathogens causing diarrhea in piglets and fattening pigs. The clinical signs of PED are vomiting, acute diarrhea, dehydration, and mortality resulting in significant economic losses and becoming a major challenge in the pig industry. PEDV possesses various crucial structural and functional proteins, which play important roles in viral structure, infection, replication, assembly, and release, as well as in escaping host innate immunity. Over the past few years, there has been progress in the study of PEDV pathogenesis, revealing the crucial role of the interaction between PEDV viral proteins and host cytokines in PEDV infection. At present, the main control measure against PEDV is vaccine immunization of sows, but the protective effect for emerging virus strains is still insufficient, and there is no ideal safe and efficient vaccine. Although scientists have persistently delved their research into the intricate structure and functionalities of the PEDV genome and viral proteins for years, the pathogenic mechanism of PEDV remains incompletely elucidated. Here, we focus on reviewing the research progress of PEDV structural and nonstructural proteins to facilitate the understanding of biological processes such as PEDV infection and pathogenesis.

## 1. Introduction

Porcine epidemic diarrhea virus (PEDV), a member of *Alphacoronavirus*, is an enveloped, single-stranded, positive-sense RNA virus that can cause porcine epidemic diarrhea (PED) [1,2]. PEDV-infected piglets display clinical signs like vomiting, diarrhea with watery stools, and dehydration, which can cause a high death rate among newborn piglets and result in substantial financial losses for the pig industry [3]. In 1971, PED was first reported in the United Kingdom [4]. After rampant transmission in many countries, PEDV became prevalent in China in 2010 [5,6]. The PEDV pandemic strains in the United States have resulted in a mortality rate of over 80% among suckling piglets [7]. Additionally, these PEDV strains have been swiftly transmitted between farms in the United States since 2013, leading to the death of more than 8 million piglets in 2014 alone and accounting for nearly 10% of all farm piglets in the country [6]. The pig industry has suffered immense economic loss as a result of the widespread transmission of PEDV. With the emergence of PEDV variants, the prevalence of PEDV has increased significantly, from 50.21% to 62.10% [8]. Recently, the pig industry in China has faced severe economic losses as a result of the emergence of highly virulent and infectious strains of PEDV [9,10].

Although herd immunization and biosafety measures are currently the most effective methods to prevent PED, the continuous evolution of PEDV, a variety of highly pathogenic strains have emerged, and the lack of mucosal immunity caused by existing PED vaccines is likely to cause immune failure, which affects the prevention and control of PEDV [10,11]. Consequently, there is an immediate requirement for a comprehensive investigation into PED and its pathogenesis, along with the production of efficient vaccines that are specifically designed for epidemic strains. Recent studies have focused on the epidemiology, diagnosis, and molecular evolution analysis of PED. However, the role of PEDV proteins in its pathogenesis still needs to be recognized [12]. Current knowledge indicates that the structural and nonstructural proteins of PEDV have a significant impact on the process of virus invasion into host cells, gene replication, transcription, translation, and immune escape [13]. To offer the most up-to-date reference for studying PEDV’s pathogenesis, we present a comprehensive review that specifically examines the roles of PEDV proteins in the development of PED. Table 1 provides a summary of the involvement of PEDV proteins in PEDV infection.

## 2. The Genome of PEDV and Its Encoded Proteins

**Figure 1:**
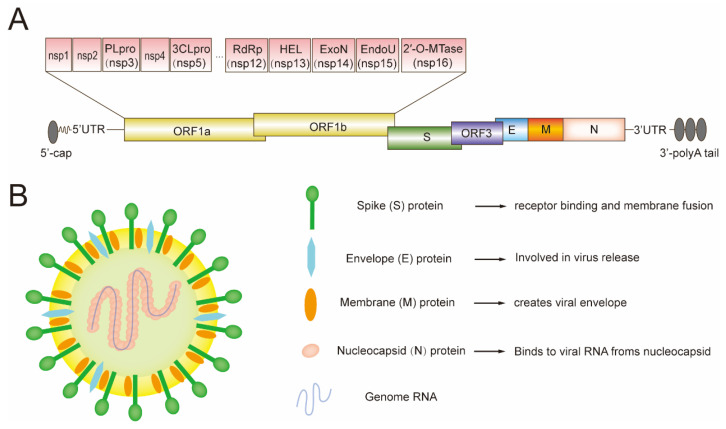
(**A**) The schematic diagram illustrates the structure of the PEDV genome. (**B**) The schematic diagram illustrates the structure of the PEDV virion.

PEDV is an enveloped, single-stranded, positive-sense RNA virus, which has a characteristic “crown-like” structure on its surface. The average size of the virus, including its spikes, ranges from 95 to 190 nm in diameter [54]. PEDV whole genome is about 28 kb long, including a 5′end cap, a 5′ untranslated region (UTR), a 3′untranslated region (UTR), a polyadenylated (poly A) tail at the 3′ end, and seven open reading frames (ORF1a, ORF1b, and ORF2–6) [55]. Among them, the polyproteins pp1a and pp1ab are encoded by ORF1a and ORF1b, respectively. The polyproteins undergo subsequent cleavage by two viral proteases, namely the papain-like protease (PLpro) and the 3C-like protease (3CLpro), which leads to the generation of 16 nonstructural proteins (nsps), designated as nsp1-nsp16. The functions of these nsps include genome replication, transcription of nonstructural proteins, and processing of viral polyproteins [56]. ORF2 and ORF4-6 encode four structural proteins, including spike protein (S, 150–220 kDa), envelope protein (E, 7 kDa), membrane protein (M, 20–30 kDa), and nucleocapsid protein (N, 58 kDa) (Figure 1) [57]. These proteins are involved in the invasion, replication, assembly, and release of PEDV, and play vital roles in PEDV survival and evasion of host immunity [12]. The PEDV accessory protein ORF3 is translated from the *ORF3* gene and is involved in the virulence of PEDV. A deficiency in ORF3 can result in a reduction in virulence [29,58].

## 3. The Replication Cycle of PEDV

**Figure 2:**
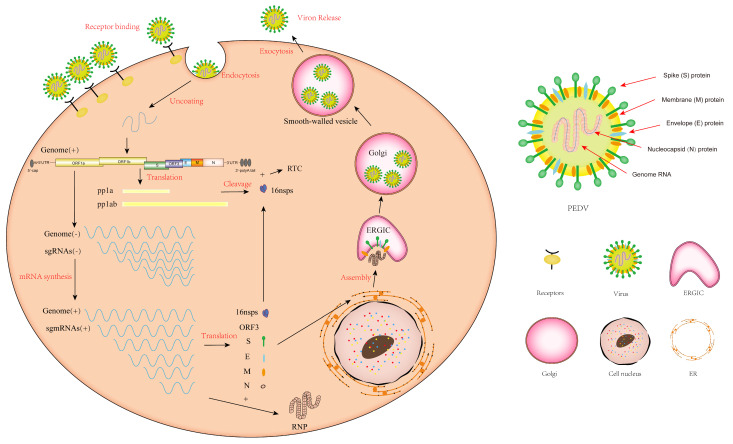
The replication cycle of PEDV.

The replication of PEDV involves multiple significant stages, including attachment and entry, translation of viral replication enzymes, genome transcription and replication, translation of structural proteins, and virion assembly and release. The replication cycle of PEDV initiates with the recognition and interaction between the S protein and its receptor, followed by fusion with the plasma membrane [59]. Following viral entry, the genome of PEDV is released, initiating the immediate translation of the two major open reading frames (ORF1a and ORF1b) within the viral genome. This translation process produces the polyproteins pp1a and pp1ab, which are subsequently cleaved into 16 nsps, including the replication and transcription complex (RTC), and was utilized to participate in subgenomic mRNA (sg mRNA) transcription and the viral genomic RNA synthesis [60]. The S, E, and M proteins, translated by sgmRNA, are inserted into the endoplasmic reticulum (ER) and immobilized within the Golgi apparatus. At the same time, the N proteins interact with newly generated genomic RNA, resulting in the formation of ribonucleoprotein (RNP) complexes. Following that, viral particles comprising RNP are assembled within the ER-Golgi intermediate compartments (ERGIC) [56,61]. Finally, the infected cells undergo cytoplasmic exocytosis to release the viral particles (Figure 2).

## 4. The Structural Proteins of PEDV

### 4.1. The Function of PEDV S Protein

Situated on the surface of PEDV particles, the spike (S) protein functions as a type I transmembrane glycoprotein. It is responsible for the formation of distinct spikes and plays a significant role in viral attachment to target cells and the production of neutralizing antibodies [62]. During virus invasion, the PEDV S attaches to the host’s receptors on the cell membrane and mediates the virus-host membrane fusion [14,15,63]. It is also the largest structural protein in PEDV, composed of 1383 amino acids, with about 150–220 kDa molecular weight [62]. Of all PEDV proteins, the S gene is a hypervariable region among different strains of PEDV. Therefore, the genetic variation of the S protein can serve as a phylogenetic indicator for assessing the genetic diversity of PEDV [64]. According to the genetic diversity of S, PEDV can be divided into two genomes, genome 1 (G1) and genome 2 (G2), which can be further divided into G1a, G1b, G2a, G2b, and G2c subgroups [65]. Among them, PEDV G1a includes the prototype strain, CV777, identified in Belgium, and all strains sharing high genetic identity with CV777 [66]. The G2 strain was highly virulent. Further homologous recombination between G1a and G2 strains led to the emergence of S-INDEL strains G1b and G2c. The G2c consisted of S-INDEL strains from America (e.g., OH851) and Europe (e.g., GER/L00862/2014) with high degrees of sequence similarity to the ZL29 strain from China, suggesting that S-INDEL strains from Europe may have originated from a common ancestor with strain ZL29 [67,68]. In the natural host, S has the capacity to elicit the production of neutralizing antibodies, so it can be used as the target for the development of neutralizing monoclonal antibodies [16]. Song et al. have generated a PED vaccine candidate by expressing the immunogenic PEDV S in an Ad5 vector, which can induce significant humoral immunity [69].

PEDV S is hydrolyzed by trypsin-like host cell proteases to produce S1 and S2 subunits [70]. Comprising two domains, the S1 subunit includes the N-terminal domain (S1 NTD, residues 21–324 based on PEDV CV777) for attaching to cellular carbohydrates, and the C-terminal domain (S1 CTD, residues 253–638) involved in binding to host [71]. The utilization of the core neutralization epitope region (COE) in the S1 region has been widespread in the development of subunit vaccines aiming to prevent viral infection [72,73]. A recent study demonstrated that the virulence of PEDV is reduced, and the antibody’s virus-neutralizing abilities are affected when the S1 region of circulating PEDV is deleted [17].

Up to now, the receptor of PEDV is still controversial; the candidates include both host glycans and host proteins. A previous study showed that PEDV S can recognize porcine aminopeptidase N (pAPN) [74] and overexpression of exogenous pAPN increases the sensitivity of PEDV to infect cells [75]. Despite this, studies have shown that PEDV is capable of infecting pAPN knockout pigs [76,77]. Further evidence indicates that the functional receptor APN is unnecessary for the infection of pigs by PEDV [78,79,80]. Many coronaviruses have been found to bind to sialic acid glycans using its S1 domain, and this also happens in PEDV [62,81,82]. Moreover, the host protein ATP1A1 could potentially serve as a functional receptor for PEDV, playing a role in PEDV attachment and co-localization with PEDV S1 protein during the initial phase of infection [83]. The PEDV S protein can induce apoptosis of Vero E6 cells, and it has been confirmed that S1 may be a key gene for PEDV-induced apoptosis [84].

The S2 subunit is related to virus fusion to host cell membrane, which contains a fusion peptide (FP, residues 891–908), two heptad repeat regions HR1 (residues 978–1117) and HR2 (residues 1274–1313), a transmembrane region (TM, residues 1328–1350), and a cytoplasmic domain (CP, residues 1351–1386) in the C-terminus [62,70,85,86]. A recent study found that the S2 protein possesses neutralizing epitopes, and the core sequence of the epitope is situated between amino acids 1261 and 1337 [87]. The infection of PEDV typically depends on the presence of trypsin. It is also a kind of tropism switching for the viruses to adapt to cultured cells if the cells are not primary or immortalized cells derived from a natural host. The available evidence indicates that three amino acid mutations, namely A605E, E633Q, and R891G, occurring in the S protein of the PEDV strain DR13att, which enable attenuated PEDV strain DR13 (DR13att) to infect Vero cells efficiently and productively, in contrast to the parental DR13 strain (DR13par). And have the potential to modify PEDV tropism by affecting the S2’ cleavage site and the RBD structure [88]. Tan et al. verified the trypsin-dependent characteristic of the S protein by comparing two PEDV strains, the trypsin-enhanced strain YN200 and the trypsin-independent strain DR13. The S protein of YN200 exhibits a stronger ability to induce syncytium formation and to be cleaved by trypsin than that of DR13. Using a full-length infectious YN200 cDNA clone confirmed that the S protein is a trypsin dependency determinant by comparison of rYN200 and rYN200-SDR13. Moreover, their findings indicate that the trypsin dependence of PEDV is predominantly controlled by the S2 subunit, as opposed to a direct trypsin cleavage site [89].

In addition, the interaction between PEDV and its host is greatly influenced by the PEDV S. In their study, Zhou et al. observed that the host membrane protein HSPA5 is capable of interacting with the PEDV S protein through its N-terminal domain, consequently controlling attachment and playing a role in the internalization process of PEDV [90]. The PEDV S contains two nearby motifs in its cytoplasmic tail (CT): a tyrosine-based motif, YxxΦ (x is any residue and Φ is a bulky hydrophobic residue: F, M, I, L, or V), and an ER retrieval signal (ERRS), KVHVQ, which might regulate the amounts of PEDV S in the endoplasmic reticulum (ER)-Golgi intermediate compartments (ERGIC) or on the cell surface [91,92,93]. Hou et al. found that the deletion of YxxØ motif and ERRS can lead to the exposure of S protein to the cell surface, promote the fusion of cell membranes, enhance the recognition of immune cells, and reduce the pathogenicity of viruses. For the intact S protein, YxxØ motif and ERRS not only help the S protein be internalized from the cell surface and escape the recognition of extracellular immune cells but also assist the S protein to migrate to the ER and Golgi apparatus to assemble mature virus particles [94]. The data offer insightful information about the different roles played by the S protein in PEDV infection.

### 4.2. The Function of PEDV E Protein

Among the structural proteins in PEDV, the envelope (E) protein is the smallest, with about 7Da molecular weight of 7000–13,000, and is distributed on the surface of the virus envelope [55]. It also exhibits strong hydrophobicity [55]. The structure of E protein includes three parts: the short N-terminal hydrophilic region, the transmembrane region containing the α helix structure with a length of about 25aa, and the long C-terminal region [95]. There is still a lack of clear understanding regarding the function of PEDV E protein. Existing research has indicated that the E protein plays a vital role in viral assembly, budding, and the host immune response.

The E protein, which is situated in the endoplasmic reticulum (ER), triggers ER stress (ERS) by upregulating GRP78 and activating nuclear factor-κB (NF-κB), resulting in enhanced expression of IL-8 and Bcl-2 [18]. In light of previous research, it has been found that the E protein has the capacity to impede the activation of RIG-I signaling, significantly inhibit the transcription of IFN-β and ISGs, and interfere with the translocation of IRF3 from the cytoplasm to the nucleus through direct interaction [19]. Further research found that the E protein initiates ERS by activating the PERK/eIF2α pathway, thereby attenuating the translation of RIG-I signaling-related antiviral proteins, ultimately inhibiting type I IFN production [96]. Moreover, the E protein can inhibit the activation of β promoters and swine leukocyte antigen II DR (SLA-DR), a crucial MHC-II molecule that plays an important role in the initiation of CD4^+^ T cell activation and antigen presentation [97,98]. The findings suggest that the E protein plays a crucial role in suppressing the immune response of the host.

Moreover, the E protein can affect the viral virulence. Li et al. found that the E protein functions as an interferon antagonist during infection and also identified it as a virulence factor of PEDV [20]. They further demonstrated that deletion of a 7-amino-acid region in the E protein (EΔaa23–aa29) could be a promising approach for the development of live attenuated vaccines against PED [20]. Host factors can also affect the virulence of PEDV through E protein. Gao et al. found that karyopherin α 2 (KPNA2) binds to the PEDV E protein, leading to its degradation through autophagy as a means to inhibit PEDV replication [99].

Furthermore, the E protein serves as a diagnostic marker that could aid in the creation of innovative serological assays and the design of vaccines that enhance protective immunity [100].

### 4.3. The Function of PEDV M Protein

The M (membrane) protein, composed of 227 amino acids, is an essential membrane glycoprotein localized throughout the cytoplasm, which can affect cell growth, cell cycle progression, and interleukin 8 (IL-8) expression [21]. The expression of the M protein in an intestinal epithelial cell (IPEC) line can cause cell cycle arrest at the S-phase through activation of the cyclin A pathway [21]. Nevertheless, the PEDV M protein neither induces ERS nor activates NF-κB [21]. The coronavirus M protein has the same structural characteristics: three TM domains with N-exo-C-endo orientation flanked by a short-glycosylated N-terminal domain on the virion surface and a long C-terminal globular domain within the virion [101,102]. The high level of conservation observed in the M gene across diverse PEDV strains makes it a promising target for the establishment of PEDV detection methods, including ELISA, RT-qPCR, and RT-PCR [103,104,105]. Among different PEDV isolates, the linear B-cell epitope (195WAFYVR200) that spans the C-terminus of the M protein is remarkably conserved. Additionally, this specific epitope has the ability to distinguish between serum samples positive for PEDV and TGEV [106]. Recent studies have utilized a 3D model to forecast four linear B-cell epitopes, with the RSVNASSGTG and KHGDYSAVSNPSALT peptides being particularly noteworthy, six discontinuous B-cell epitopes, forty weak binding, and fourteen strong binding T-cell epitopes in the CV777 M protein [107].

The M protein serves as an essential structural protein in the processes of viral infection, replication, and assembly. Additionally, it can activate the immune response of the host for protection, although the precise mechanisms behind these functions are still not fully understood. The PEDV M protein has been recognized as an interferon antagonist [22]. Recent studies found that the 1–55 region of M protein is essential for disrupting IFN regulatory factor 7 (IRF7) function, suppressing the expression of type I IFN and various antiviral ISGs, enhancing viral replication, and determining the antagonistic mechanism used by M protein to target IFN [108]. Wang et al. found that M protein interacts with eukaryotic translation initiation factor 3L (eIF3L) during PEDV infection, downregulates the expression of eIF3L, and significantly increases virus replication [109]. Moreover, the M protein directly interacts with HSP70, thereby facilitating PEDV replication [110]. These observations indicate that M protein has the potential to serve as a candidate for the development of a differential diagnostic test. Additionally, it could be a valuable target in the design of multi-epitope vaccines and novel therapeutic approaches aimed at activating protective cellular mechanisms against PEDV.

### 4.4. The Function of PEDV N Protein

The N protein is a nucleocapsid protein composed of 1383 amino acids, which is highly conserved and undergoes alkaline phosphorylation. It has a relative molecular weight of about 56 kDa [111]. The N protein plays a crucial role in multiple aspects of the virus’s life cycle, such as controlling viral RNA synthesis, packaging viral RNA into the helical nucleocapsid, and assembling viral particles [112,113]. A recent study has discovered that the PEDV SH strain N protein contains a distinctive deletion of 12 amino acids (aa 399–410), which includes an antigenic epitope. However, this deletion does not impact the immunogenicity or pathogenicity [114]. The N protein of PEDV has three conserved domains: N-terminal domain (NTD), linker region (LKR), and C-terminal domain (CTD). In addition, there is an intrinsically disordered region (IDR) at both ends [115]. The N protein is highly expressed during all stages of PEDV infection. Therefore, identifying the N protein enables the assessment of PEDV replication, rendering it useful for the early detection of PEDV infection [116,117]. The cytoplasm serves as the main location for the N protein; however, it can also be detected within the nucleolus. It possesses a nuclear localization signal (NLS) motif pat7 (261PKKNKSR267) and is actively transferred to the nucleolus during the time span of PEDV infection [118]. According to the study conducted by Shi et al., the translocation of N protein from the cytoplasm to the nucleolus is not reliant on the shuttle protein nucleolar phosphoprotein nucleophosmin (NPM1). However, N protein can interact with NPM1 to inhibit its proteolytic cleavage and improve cell survival, ultimately facilitating PEDV replication [119]. N protein has an RNA binding domain (RBD) that binds to RNA packaging signal (PS) and can associate with genomic RNA to form a ribonucleoprotein (RNP) complex, which participates in the replication and transcription process of the virus. It can also bind to M protein to promote the assembly of viral particles [23,115]. The N protein NLS (S71NWHFYYLGTGPHADLRYRT90) can interact with p53 and activate the p53-DREAM pathway, leading to S-phase arrest and finally promoting virus replication [24].

The N protein also actively participates in regulating host innate antiviral responses and regulates IFN signal transduction. The N protein has the ability to specifically impede the nuclear translocation of NF-κB, which relies on type III IFN while leaving the expression of type I or type II IFN induced by polyinosinic-polycytidylic acid (poly(I:C)) unaffected in IPEC-J2 cells [25]. During the interaction with host cells, the PEDV N protein exerts an inhibitory effect on cell proliferation by extending the duration of the S-phase cell cycle [26]. Xu et al. studied the subcellular localization and the effect of PEDV N protein on cell growth, cell cycle progression, cell survival, and IL-8 expression [26]. The N protein located in the ER can induce ERS, inhibit cell growth, and upregulate the expression of IL-8 in IPEC cells. The NLS sequence of the PEDV N protein is located within its amino acid region 71–90, with R87 and R89 being crucial for its biological function. Additionally, two nuclear export signals (NES) are at amino acids 221–236 and 325–364. However, only the NES within the 325–364 region is involved in the full-length N protein’s functionality [26]. Ding et al. discovered that the N protein also hinders the production of IFN-β induced by the Sendai virus, the expression of the IFN-stimulated gene, as well as the activation of the transcription factors IFN regulatory factor 3 (IRF3) and NF-κB [27]. Moreover, by engaging in a direct interaction with TBK1, the N protein disrupts the TBK1-IRF3 association, resulting in the inhibition of both IRF3 activation and type I IFN production [27]. Likewise, Cao et al. observed that PEDV-infection in IECs impedes the dsRNA-induced IFN-β induction by interfering with IRF-3, which is associated with the RIG-I-mediated signaling pathway and its adapter molecule, IFN-β promoter stimulator 1 (IPS-1) [28]. However, it is not known whether it is caused by the N protein.

Furthermore, the N protein plays a crucial role in the interaction between the coronavirus and its host, as well as in the pathogenesis. The PEDV N protein serves as a multifunctional protein, playing a crucial role in facilitating PEDV replication by targeting specific host cytokines. For example, the N protein promotes the cyclization of viral mRNA through binding to PABPC1 and eIF4F proteins, thus promoting viral transcription and facilitating viral replication [120]. PEDV N protein can interact with TRIM28 to induce mitophagy, leading to inhibition of the JAK-STAT1 pathway to promote PEDV replication [121]. On the other hand, the host can use host cytokines to hijack the N protein and then degrade it to inhibit the replication of PEDV. The E3 ubiquitin ligase MARCH8 facilitates the interaction between EGR1 and the PEDV N protein, resulting in N protein ubiquitination and degradation. Additionally, EGR1 directly binds to the IRAV promoter, leading to the upregulation of IRAV expression and subsequent suppression of PEDV replication [122]. PTBP1 facilitates the recruitment of MARCH8, an E3 ubiquitin ligase, and NDP52, a cargo receptor, for the catalysis and degradation of N protein via selective autophagy, thereby inhibiting PEDV replication [123]. The activation of autophagy by HNRNPA1 promotes the degradation of PEDV N, while the N protein-mediated autophagy pathway triggered by PEDV infection also results in the degradation of HNRNPA1 protein [124]. PRPF19 can recruit the E3 ubiquitin ligase MARCH8 to the N protein for ubiquitination, and the ubiquitinated N protein is recognized by the NDP52 and transported to autolysosomes, thus inhibiting virus replication [125]. HnRNP K inhibits PEDV replication through autophagic degradation of viral N protein and upregulation of MyD88 expression, leading to IFN-1 production [126]. RALY can degrade the N protein through a RALY-MARCH8-NDP52-autophagosome pathway to suppress PEDV replication [127]. FUBP3, TARDBP, and BST2 are capable of initiating the degradation of the N protein via the MARCH8-NDP52-autophagosome pathway, resulting in the induction of IFN-I production, in an effort to hinder the replication of PEDV [128,129,130]. TRIM21 selectively directs the degradation of the N protein through a proteasome-dependent mechanism, resulting in the inhibition of PEDV replication [131]. Hence, the enhanced replication of PEDV in Vero E6 cells with overexpression of N protein could potentially be attributed to the suppression of host immune response induced by N protein [132].

In summary, the N protein performs various functions, while further exploration is required to fully comprehend its underlying mechanism. These data have improved our knowledge regarding the function of PEDV N protein.

## 5. The Accessory Protein of PEDV

ORF3 serves as the sole accessory protein in PEDV, situated between S protein and E protein. It consists of 224 amino acids. Based on bioinformatic predictions, it has been determined that the PEDV ORF3 protein is a viroporin, possessing four transmembrane domains. Furthermore, it acts as a potassium ion channel protein, regulating the release of the virion [133]. When PEDV is adapted to growth in cell culture, the protein is vulnerable to deletion or mutation. [134,135]. Research findings indicate that viruses lacking ORF3 or with a truncated ORF3 demonstrate complete attenuation in vivo, enabling us to distinguish cell-adapted and wild-type PEDV strains [136,137]. Thus, the ORF3 gene holds promise as a potential instrument for conducting epidemiological research on PEDV infections [138]. Typically, with the exception of CH/GSJIII/07, Chinese PEDV field strains exhibit a single ORF of 672 or 675 nucleotides in their ORF3 genes [139]. In cell-adapted strains of PEDV, such as DR13, CV777, KPED-9, and P-5V, deletion of either 51 or 49 nucleotides within the ORF3 gene has been observed due to continuous in vitro passage. This deletion results in premature termination of translation and a frameshift in the reading frame [133,140]. However, it has been confirmed that PEDV field strains discovered in China, which possess a naturally occurring truncated ORF3 gene, exhibit high virulence, resulting in severe clinical signs and elevated mortality rates among suckling piglets [141,142]. The study conducted by Jiang et al. revealed that the ORF3-null recombinant PEDV exhibited larger plaque size and syncytia phenotypes than the wild-type ancestor. Additionally, the ORF3-null virus demonstrates a relatively rapid growth phenotype [143]. According to recent studies, recombinant PEDV with truncated ORF3 has a minimal effect on pathogenicity. This suggests that ORF3 may not be necessary for PEDV propagation in vitro [144]. The findings of these studies indicate an enigmatic role of the ORF3 protein in the pathogenicity of PEDV.

Apart from its impact on PEDV virulence, the ORF3 protein also has multiple functions in cellular processes. During virus infection, there is a potential interaction between the full-length and naturally truncated ORF3 proteins with the S protein. This suggests that the ORF3 protein could potentially support the binding of the S protein to cell receptors, thereby enhancing virus infection in cells [145]. ORF3, like the M protein, can arrest cells at the S-phase and facilitate the formation of vesicles, and attenuated strain expressing a truncated ORF3 is prone to replicate faster [29]. The ORF3 protein, localized in the ER, triggers the ERS response by increasing the expression of GRP78 protein and activating the PERK-eIF2α signaling pathway. This activation leads to autophagy but has no impact on apoptosis [30]. Similarly, Chen et al. also confirmed that overexpression of ORF3 protein does not induce apoptosis [84]. However, Si et al. found that compared to artificially rescued PEDV recombinant virus strains carrying full-length *ORF3* gene (rPEDV-ORF3wt, rPEDV-ORF3CV777, and rPEDV-ORF3NY), PEDV recombinant virus strains without *ORF3* gene (rPEDV-ΔORF3) can cause more severe cytopathic effect (CPE); ORF3 protein can promote virus replication by suppressing cellular apoptosis induced by PEDV [58]. Moreover, the study uncovered that the PEDV ORF3 hinders virus replication through its interaction with the cellular vacuolar protein-sorting-associated protein 36 (VPS36) [146]. Furthermore, the ORF3 protein is involved in the PEDV immune evasion strategy. The ORF3 protein was found to inhibit the production of proinflammatory cytokines (IL-6) in host cells by blocking the NF-κB pathway, enabling PEDV to evade the innate immune response [31]. Kaewborisuth et al. discovered that the ORF3 protein can enhance the activity of the IKBKB-mediated NF-κB promoter, inhibit the activation of the IKBKB-mediated IFN-β promoter, and suppress the production and induction of type I IFN by poly (I:C) [32].

Collectively, these studies highlight the versatility of the ORF3 gene/protein, which is implicated in diverse cellular processes and is pivotal for PEDV infection, packaging, release, PEDV-host interaction, and preservation of host immunity.

## 6. The Nonstructural Proteins of PEDV

The poly-proteins pp1a and pp1b are encoded by the partially overlapping 5’-terminal of the PEDV genome and can undergo cleavage, producing 16 mature replicase proteins known as nonstructural proteins 1–16 (nsp1–16) [147,148]. These nonstructural proteins can function independently and have the capacity to influence one another. The nonstructural proteins encoded by PEDV are mainly involved in the compilation of the viral replication enzyme complex and the synthesis of viral RNA. They are expressed during viral replication but do not appear in mature viral particles.

The nonstructural protein 1 (nsp1) comprises 110 amino acids and has high nucleotide similarity to the nsp1 of β-coronavirus; the conserved hydrophobic amino acid sequence plays a crucial role in preserving the protein’s structure, thus implying a possible similarity in structure and function. Niu et al. discovered that a genetically modified PEDV mutant with nsp1 N93A and N95A mutations demonstrates partial attenuation and retains immunogenicity in neonatal pigs. These findings imply that targeting nsp1 N93 and N95 could be a promising strategy for the development of live attenuated vaccines. Furthermore, it could potentially serve as a promising candidate for vaccine development targeting other β-coronaviruses [149]. Among all the proteins encoded by PEDV, nsp1 is an effective IFN antagonist, has the most obvious effect on disrupting host gene expression, and can suppress or evade the innate immune response and facilitate viral replication [33]. Consequently, nsp1 is considered a crucial factor for virulence and a potential target for vaccine development. The nsp1 can promote proteasome-mediated degradation of cAMP response element binding protein (CBP), making IRF3 unable to bind to CBP after phosphorylation into the nucleus, hindering the formation of enhancer complex, thereby inhibiting type I IFN and proinflammatory cytokine production [22]. It can also block the nuclear translocation of IRF1, leading to a decrease in peroxisomes and consequently suppressing the IRF1-mediated type III IFNs [34]. In addition, nsp1 hinders the phosphorylation and degradation of IκBα, thus impeding the activation of p65 and suppressing the NF-κB activity mediated by the positive regulatory domain (PRD) II in vitro [35]. The nsp1 can inhibit Complement component 3 (C3) by inhibiting CCAAT/enhancer-binding protein β (C/EBP-β) phosphorylation via amino acid residue 50 [150]. Additional research on the structure and biochemical properties of the PEDV nsp1 has unveiled that its three motifs (aa 67 to 71, 78 to 85, and 103 to 110) form a stable, functional region capable of suppressing the production of host proteins, including IFN [151,152].

Similarly, other nonstructural proteins of PEDV have been shown to play a role in the process of regulating viral genome replication, transcription, and anti-host innate immune response. Through the K48-linked ubiquitin-proteasome pathway, the nonstructural protein 2 (nsp2) can interact with FBXW7, leading to its degradation and ultimately promoting the replication of PEDV [36].

Nonstructural protein 3 (nsp3) is the largest transmembrane protein encoded by PEDV and contains two papain-like protease (PLpro) domains (PLP1 and PLP2). PLP2 has deubiquitinating enzymes (DUB) activity, which can regulate the de-ubiquitination of RIG-I and stimulator of interferon gene (STING) thus inhibiting type I IFN expression [37]. Similar findings have been found in other coronaviruses. SARS-CoV-PLpro inhibits STING-mediated activation of IRF-3 nuclear translocation and induction of IRF-3 dependent promoters and blocks STING dimer formation [38]. The SCoV2-PLpro selectively targets the regulators of the RLR signaling pathway through both deubiquitination-dependent and -independent mechanisms. The inhibitory effect of SCoV2-PLpro on RIG-I and TBK1 is connected to its DUB function, whereas its antagonistic effect on MAVS, TRAF3, TRAF6, and IRF3 is not reliant on the DUB activity [153]. 

Unlike the nsp3, the nonstructural protein 4 (nsp4) can promote the expression of pro-inflammatory cytokines and chemokines, such as IL-1α, IL-1β, TNF-α, CCL2, CCL5, and CXCL8, by upregulating the NF-κB pathway to inhibit PEDV replication during viral infection [39]. 

The nonstructural protein 5 (nsp5), also known as 3C-like protease (3CLpro), can cleave proproteins into nsp5 and nsp16, leading to their maturation and subsequent involvement in multiple stages of virus replication [40]. Due to its conserved structure and catalytic mechanism, the coronavirus 3CLpro assumes a crucial role in viral polyprotein processing, making it a highly desirable candidate for antiviral drug targeting. The research conducted by Ye et al. demonstrated that the inhibitor GC376, which targets 3Cpro or 3CLpro of viruses in the picornavirus-like supercluster, can effectively hinder viral replication by binding to the catalytic pocket of PEDV 3CLpro [41]. Additionally, the nsp5 protein functions as an interferon antagonist by specifically cleaving the NF-κB essential modulator (NEMO) at glutamine 231 (Q231). NEMO plays a critical role in linking interferon-regulatory factor and NF-κB activation [154]. The same function is also found in nsp5 of PDCoV, which can inhibit SEV-induced IFN-β production by targeting the NEMO [155]. It is worth noting that the PLpro domain of nsp3 and the 3CLpro domain of nsp5 have been confirmed to possess a viral replication-promoting effect, which is associated with their role in processing pp1a and pp1ab polyprotein precursors into nsps. In addition, nsp5 of PDCoV can also cleave STAT2 but not Janus kinase 1 (JAK1), tyrosine kinase 2 (TYK2), STAT1, and interferon regulatory factor 9 (IRF9) to antagonize the signal transduction downstream of the IFN receptor [156]. However, whether there is a similar mechanism of action of nsp5 in PEDV-infected cells still needs further study.

Recent evidence indicates that the nonstructural protein 6 (nsp6) can induce autophagy via the PI3K/Akt/mTOR signaling pathway [157].

The nonstructural protein 7 (nsp7), consisting of 83 amino acids, is a polypeptide that is predicted to have four helices. It is conserved within the *Coronaviridae* family and is essential for viral replication. The nsp7, in combination with nsp8 and nsp12, forms a viral replication and transcription complex that is crucial for viral replication [158]. Nonetheless, apart from its role as the “mortar” that stabilizes the nsp7/nsp8 complex structure, there is limited understanding of its functions [159]. The nsp7 can interact with the DNA binding domain of STAT1/STAT2, leading to the interaction between karyopherin α1 (KPNA1) and STAT1. As a result, the nuclear transport of ISGF3 is blocked and type I IFN signaling pathway is inhibited [42]. Subsequent investigations revealed that nsp7 has the ability to selectively bind to the caspase activation and recruitment domains (CARDs) of melanoma differentiation-associated gene 5 (MDA5). This interaction effectively hinders the association between MDA5 and the protein phosphatase 1 (PP1) catalytic subunits (PP1α and PP1γ), resulting in the inhibition of MDA5 S828 dephosphorylation and subsequent suppression of the type I IFN signaling pathway [43].

Additionally, it has been demonstrated that nsp8 can inhibit type III IFN activities by decreasing IRF1 promoter activities in vitro, indicating that nsp8 may function as immunosuppressive factors. However, the precise mechanism of innate immunosuppression caused by nsp8 is still unidentified [22].

The nonstructural protein 9 (nsp9) is a vital element in coronavirus replication as an essential RNA binding protein. It is characterized by seven antiparallel β-strands and one α-helix strand [160]. The interactions between nsp9 and RNA potentially play a role in stabilizing viral RNAs during viral replication or transcription. Furthermore, the dimerization of nsp9 and its positively charged surface can amplify this interaction [60,161]. The nsp9 protein could potentially be utilized as a promising target for the development of effective diagnostic tools or vaccines against PEDV. Liu et al. discovered that nsp9 has the ability to activate the swine immune system, leading to the development of humoral immunity. Moreover, the resulting antibody exhibits inhibitory effects on PEDV proliferation in Vero cells [162]. Furthermore, the nsp9 has the capacity to increase the expression of H2BE, thereby suppressing ERS-induced apoptosis and promoting viral replication. This process is reliant on the N-terminal domain of H2BE (amino acids 1–28) [44].

Nonstructural protein 10 (nsp10) plays a vital role as a regulator in viral RNA synthesis by enhancing the activities of nsp14 ExoN and nsp16 2′-O-MTase, which is indispensable for viral replication [45,163]. Recent research found that nsp10 can upregulate the expression of IL-2, IL-4, IL-10, TNF-α, and IFN-γ to induce the cellular immune response in mice and inhibit PEDV replication [46]. It is suggested that nsp10 exhibits a high level of immunogenicity, making it a suitable target for the development of antiviral drugs or a potential candidate for the rapid diagnosis of PEDV infection.

The nonstructural protein 12 (nsp12) is an RNA-dependent RNA polymerase (RdRp), which is a key replication enzyme for viral replication [47]. At present, there are few studies on PEDV nsp12.

Nonstructural protein 13 (nsp13) functions as a nucleic acid helicase/NTPase and is crucial for viral gene transcription and replication [164]. Furthermore, the nsp13 exhibits robust ATPase functionality, capable of hydrolyzing adenosine triphosphate (ATP) in the presence of Mg^2+^ and Mn^2+^. Moreover, it has the ability to bind to both double-stranded RNA (dsRNA) and double-stranded DNA (dsDNA) and effectively unwind the substrates in a 5’-to-3’ orientation, utilizing the energy derived from the hydrolysis of all nucleoside triphosphates (NTPs). The role of lysine 289 (K289) in PEDV nsp13 is crucial for its ATPase and helicase functions [164]. According to recent studies, nsp13 is implicated in the immune evasion of PEDV. It has the potential to impact the NF-κB canonical pathway and enhance the expression of DNA methyltransferase 3b (DNMT3b) protein through the facilitation of p65 protein binding to chromatin. Consequently, this leads to abnormal methylation of the neonatal Fc receptor (FcRn) promoter, ultimately inhibiting the bidirectional transport of IgG. In addition, the essential domain for FcRn inhibition is within the core region of nsp13 (230~597 aa) [48].

CoV nonstructural protein 14 (nsp14) has 3′-to-5′ exoribonuclease (ExoN) activity [165], which is important for the fidelity of CoV RNA genome replication, and N7-methyltransferase (N7-MTase) activity [166], which is also crucial for viral transcription and translation. The research conducted by Niu et al. revealed that recombinant PEDV variants carrying mutations at the crucial functional sites (E191A) within nsp14-ExoN exhibit either lethality or genetic instability [167]. Abolishing N7-MTase activity has lethal consequences for PEDV, and altering a single amino acid (D350A) in nsp14 can maintain 25% of N7-MTase activity and ensure viability. In addition, N-7 MTase-deficient mutant induces increased secretion of type I and III IFNs [168]. Nsp14 can inhibit the activity of GRP78 promotor, and its N7-MTase domain is necessary for this role. This result suggests that PEDV possesses the potential to antagonize ER stress via nsp14 [49]. Nsp14 can also suppress the host antiviral response. Nsp14 is an NF-κB pathway antagonist that remarkably decreases SeV-, poly (I:C)-, and TNF-α-induced NF-κB activation [50]. Furthermore, it demonstrates the ability to suppress the expression of proinflammatory cytokines, inhibit the phosphorylation of IKKs by interacting with IKKs and p65, and impede the phosphorylation and nuclear import of p65 induced by TNF-α [50].

Nonstructural protein 15 functions as an endoribonuclease (EndoU) and is essential for viral replication and evasion of the host’s antiviral defense mechanisms [169]. The EndoU activity of nsp15 relies on H226, H241, and K282. The formation of stress granules (SGs) in host cells is impeded by the nsp15 protein by reducing the accumulation of viral double-stranded RNA and isolating the key elements of SGs [170]. The nsp15 not only inhibits the activities of IFN-β and IRF3 promoters but also suppresses the activity of type III IFN [22,34]. Moreover, it has the ability to antagonize IFNs and obstruct the chemokine system in order to establish a suitable intracellular milieu for viral replication. Nsp15 can subvert the IFN response by suppressing TBK1/IRF3 dependent-EndoU activity to facilitate PEDV replication [51]. A recent study found that nsp15 can inhibit several genes involved in immune responses and inflammation, such as CCL5, CXCL8, CXCL10, OAS, MXs, STAT1, and IRF9 [52].

Among coronaviruses, the nonstructural protein 16 (nsp16) shows a highly conserved 2′O-methyltransferase activity [171]. PEDV nsp16 is dependent on the KDKE tetrad, which has the ability to suppress the functions of RIG-I/MDA5-mediated IFN-β and ISRE, thus facilitating virus replication. Moreover, nsp10 was discovered to enhance the suppressive impact of nsp16 on IFN-β production [172]. The decrease in PEDV 2′-O-MTase activity leads to a reduction in the virus virulence in piglets and triggers enhanced type I and type III IFN responses in IPEC-DQ cells [53]. This suggests that nsp16 could serve as a potential target for the development of PEDV vaccines.

It is worth mentioning that nsp14-nsp16 contributes to the RNA cap formation of CoVs. Under the influence of a cis-element present in viral genome RNA, the virally encoded RdRP synthesizes nascent genome and subgenome RNA. Subsequently, nsp13 hydrolyzes the RNA, resulting in the production of ppN-RNA [173]. Following this, the ppN-RNA is capped through the catalytic activity of an unidentified CoV capping enzyme, leading to the synthesis of GpppN-RNA. The cap structure can be further methylated at the G-N-7 position by CoV nsp14. Lastly, the complex comprising CoV nsp16 and its stimulatory factor nsp10 functions as a 2’-O-MTase, thereby raising the cap 0 to cap 1 structure [163,174,175]. Of note, the crystal structures of various cap-forming enzymes in SARS-CoV-1, such as nsp14, nsp16, and the nsp16-nsp10 complex, have been accurately determined. Consequently, this has facilitated the precise identification of their catalytic sites at the amino acid level [176,177].

Over the past few years, a growing body of research has demonstrated that numerous nonstructural proteins of PEDV possess the ability to suppress the innate immune response of the host, thereby facilitating the rapid proliferation and dissemination of invading viruses. Therefore, it is reasonable to suggest that these proteins may enhance the effectiveness of vaccines, and the signaling pathways they impact could be investigated as potential targets for the development of a PEDV vaccine, provided that relevant concerns are appropriately addressed.

## 7. Conclusions

Given the persistent mutability of the PEDV virus, it is conceivable that the current PEDV vaccine has failed to mitigate the PEDV epidemic [10]. Consequently, studying the mechanism of PEDV proteins can greatly contribute to the development of new PEDV vaccines and the implementation of more efficient strategies for the prevention and control of PED. PEDV vaccines are mainly divided into inactivated vaccine, attenuated vaccine, and subunit vaccine [178]. Among these vaccines, both inactivated and attenuated live vaccines, as whole-virus vaccines, may experience a partial or complete decline in their capacity to withstand the challenge of parental strains, even if the strains’ virulence is weakened through repeated passages. Hence, certain researchers have initiated the alteration of PEDV strains using reverse gene operating systems in order to create live attenuated vaccines that possess high immunogenicity while exhibiting reduced virulence [86]. The structural protein S [94], accessory protein ORF3 [136], and the nonstructural proteins nsp1 [179], nsp14 [168], nsp15 [179], and nsp16 [53,179] can be considered as potential targets for the development of PEDV attenuated live vaccines. This is attributed to the significant reduction in viral virulence observed when specific mutations occur in these genes. In summary, employing reverse gene technology to introduce virulence gene mutations and inactivate interferon antagonists proves to be a successful approach in developing highly efficacious live attenuated PEDV vaccines.

The subunit vaccine, in contrast to the entire virus vaccine, boasts notable advantages such as enhanced safety, reduced propensity for spread, and minimal risk of reversion. The PEDV S protein is widely recognized as a crucial target for stimulating immune response and generating neutralizing antibodies, making it the primary focus for subunit vaccine development [180,181]. Nevertheless, despite the presence of epitopes in N and M proteins, the development of subunit vaccines utilizing PEDV N and M proteins remains unreported [106,182]. Moreover, additional verification is required to determine whether subunit vaccines utilizing PEDV nonstructural proteins can effectively induce protective responses.

Considering the significant influence and danger presented by PEDV variant strains, the advancement of anti-PEDV drugs and the exploration of associated antiviral mechanisms hold great significance. Research shows that antiviral drugs such as prenylated phenolic compounds [183], magnolol [184], bis-benzylisoquinoline alkaloids [185], Ginkgo biloba exocarp [186], Aloe extract [187], glycyrrhizin [188], and quercetin 7-rhamnoside [189,190] can be used as potential anti-PEDV drugs. Despite the effectiveness of anti-PEDV drugs, their adoption in clinical practice remains limited due to their prohibitive cost and inability to achieve the desired antiviral effect. Currently, there is a lack of commercially available anti-PEDV drugs, and there have been no reports of drugs specifically targeting PEDV protein.

Furthermore, it is also particularly important to study the relationship between host and viral proteins and explore the immune escape mechanism of PEDV infection for the prevention and control of PEDV. Host cells can establish intricate signaling networks to identify, control, and eliminate invading viruses. However, these antiviral pathways are frequently evaded, inhibited, or disrupted by PEDV countermeasures. The PEDV demonstrates the capability to utilize host factors to promote its own replication. Consequently, the study of the interplay between host and viral proteins, along with the investigation of the immune evasion mechanism in PEDV infection, assumes paramount significance. This paper provides a summary of the role of PEDV protein in viral cell invasion, viral replication and transcription, host immune protection, and the mechanism of interaction with the host. It is anticipated that it will offer fresh insights into the development of PEDV vaccines, targeted pharmaceuticals, and the prevention and control measures for PED.

## Figures and Tables

**Table 1:** The viral proteins involved in PEDV infection.

Classification	Viral Protein	Role in PEDV Infection	References
Structural proteins	S	Essential for viral entry, induces neutralizing antibodies, virulence, induces apoptosis	[14,15,16,17]
	E	Initiates ERS, activates NF-κB, inhibits IFN-β, ISGs, virulence	[18,19,20]
	M	Induce cell cycle arrest at the S-phase, acts as IFN antagonist	[21,22]
	N	Form ribonucleoprotein complex, induces S-phase arrest, induces ERS, upregulates the expression of IL-8, inhibits IFN-β and IFN-λ production	[23,24,25,26,27,28]
Accessory protein	ORF3	Arrests cells at the S-phase, triggers ERS, induces autophagy, inhibits IL-6 and IL-8 productions, upregulates IKBKB-meditated NF-κB promoter, downregulates IFN-β promoter	[29,30,31,32]
Nonstructural proteins	Nsp1	Acts as IFN antagonist, induces virulence, inhibits proinflammatory cytokine production, inhibits NF-κB activity	[22,33,34,35]
	Nsp2	Promotes the replication of PEDV	[36]
	Nsp3	PLpro, regulates the deubiquitination of RIG-I and STING, inhibits IFN-β and IFN-λ1	[37,38]
	Nsp4	Upregulates pro-inflammatory cytokines and chemokines expression (IL-1α, IL-1β, TNF-α, CCL2, CCL5, and CXCL8）	[39]
	Nsp5	3C-like protease，IFN antagonist	[40,41]
	Nsp7	Inhibits type I IFN	[42,43]
	Nsp8	Inhibits type III IFN	[22]
	Nsp9	Inhibits ERS-mediated apoptosis	[44]
	Nsp10	Essential for viral replication, upregulates IL-2, IL-4, IL-10, TNF-α, and IFN-γ	[45,46]
	Nsp12	RdRp, viral replication	[47]
	Nsp13	HEL, inhibits bidirectional IgG transport by FcRn	[48]
	Nsp14	ExoN, suppresses ER stress-induced GRP78, acts as NF-κB pathway antagonist, downregulates pro-inflammatory cytokines	[49,50]
	Nsp15	EndoU, inhibits IFN-β and IRF3, downregulates CCL5, CXCL8, CXCL10, OAS, MXs, STAT1, and IRF9	[51,52]
	Nsp16	2′-O-MTase, downregulates the activities of RIG-I/MDA5-mediated IFN-β and ISRE	[53]
